# Meeting a Fork in the Road: An Interview with Tom Cech

**DOI:** 10.1371/journal.pgen.0010076

**Published:** 2005-12-30

**Authors:** Jane Gitschier

Long before his meteoric rise to a Nobel Prize at age 42 and to the presidency of the Howard Hughes Medical Institute (HHMI) a decade later, Tom Cech was just another guy working down the hall from me. In the mid 1970s, our lives intersected at the Massachusetts Institute of Technology (MIT) when he was a postdoc and I a graduate student. I recall a lanky midwesterner, modest and likeable, earnestly working on a project that didn't seem too exciting, or certainly didn't seem to be the stuff of scientific breakthrough, prizes, or fame.

I took advantage of our brief shared history to enlist Tom for an interview. We set up a video conference: Tom, nursing what appeared to be a green bottle of Perrier at the HHMI headquarters, and I, armed with a re-heated cup of coffee in the pristine confines of the HHMI conference center at the University of California San Francisco. The video transmission suffered from episodes of stutter and delay, with Tom's voice and image out of sync, disconcertingly causing Tom to look like a man on a lunar mission in an Apollo spacecraft, rather than safely ensconced in Chevy Chase.

We got the ball rolling with a bit of reminiscing about our experience 30 years ago at MIT.

**Jane Gitschier:** When I came to MIT in September of 1975, I recall being thrust into an exhilarating atmosphere. The first seminar of the year, called together hastily, was by Phil Sharp, who had just returned from Cold Spring Harbor to describe RNA splicing for the first time. One month later, champagne was flowing in the hallways because David Baltimore was awarded a Nobel Prize. It was something!

**Tom Cech:** I had the same experience. We were in the old-fashioned biology building, 16–56, but it was a very exciting place. My benchmate [in Mary Lou Pardue's lab] was Joan Ruderman, and Matt Scott and Al Spradling were in the lab at the same time. It was a very small lab, but half the people ended up getting elected to the National Academy of Sciences. There was a very high level of scholarly interest, good critical thinking, and a lot of excitement about science.

**Gitschier:** I recall you studying psoralen cross-linking. What were you really up to?

**Cech:** I was looking for a way to freeze nucleic acid structures in vivo, and then to pull them out without rearranging them. I was particularly interested in alternative secondary structures of DNA, cruciform structures, and I was doing almost exclusively electron microscopy. I did that for a long time! I was trained in the Cal Tech/Norm Davidson/Phil Sharp/Ron Davis tradition of quantitative DNA electron microscopy, so you always had to measure hundreds of molecules before you believed what you were seeing in the EM [electron microscope].

Most DNA molecules look very boring because they are just a double helix. But if it's branched, or if it's a replication form or a Holliday junction or some alternative form, it [psoralen cross-linking] can be a useful tool. But then John Hearst's lab found that information about where the nucleosomes were located on the DNA was also preserved because the regions that were in the linkers between nucleosomes were much more accessible to cross-linking than the regions wrapped around the histones.

I built a zapper. I bought a high-intensity mercury vapor lamp, the kind used in streetlights, and I set it all up myself! I was amazed that no one got killed because I never had any training. I built this thing with the shield and the switch and the temperature-controlled reaction chamber. You don't realize how bright and hot these streetlights are till you get one a foot away. There is an immense flux of light coming out of there. And that's the wavelength of radiation that activates the psoralen cross-linking. That thing may still be there at MIT.

**Gitschier:** So you moved to Boulder, left the cross-linking, and moved on to ribosomal RNA genes.

**Cech:** The main thing I decided was to do something completely different when I started my faculty position.

**Gitschier:** That seems unusual; most people capitalize on the progress they've made as a postdoc.

**Cech:** I think it was a little less unusual then than now. I wanted to look at the chromatin structure of a transcription unit. People could clone DNA by then, but they couldn't clone DNA with its natural histone and nonhistone proteins associated with it in the right place. Tetrahymena had 10,000 identical copies of the ribosomal gene. I thought that one could isolate the gene in its chromatin state and look for regulation of transcription. It was a fine idea—it never worked.

**Gitschier:** But fortunately, something else worked!

**Cech:** But fortunately, the gene had an intron, which it wasn't even supposed to have! As you mentioned, this was right after Phil Sharp and Cold Spring Harbor had discovered RNA splicing. By the time I was an assistant professor, there were 100 examples of eukaryotic introns, but no information about splicing mechanisms. In fact, people were just starting to come to grips with the fact that it was splicing rather than some kind of transcriptional jumping.

**Gitschier:** I remember that debate.

**Cech:** There were several papers, including one by Shirley Tilghman and Phil Leder, showing that there was a precursor RNA that was then processed into spliced RNA, so it looked like splicing was operating at the RNA level. But there was only one lab, John Abelson's in Southern California, studying yeast splicing, which had much of a handle on splicing in vitro.

So when we found that the intron in our ribosomal genes was being spliced, there was a fork in the road. We could either say, “We're not funded to do splicing research by the NIH [National Institutes of Health]; we're funded to do transcriptional regulation, so let's just report that intron and leave that for someone else to follow up,” or we could follow this splicing angle. We tried to do both for a while, but of course the splicing became more interesting.

**Gitschier:** Tell me more about that discovery.

**Cech:** It was during a period of about a year when I was insisting that there had to be a protein enzyme stuck to the RNA that was doing the splicing, and we were trying to shake it off so that we could get back to studying the splicing protein. Boiling the RNA, adding ionic detergent, boiling in the presence of detergents, adding proteases—all had no effect. We were getting more and more desperate!

As time went on, it seemed less and less likely that there could be a protein, but if we announced that the RNA was self-splicing, no one was going to believe us! So we then turned to recombinant DNA, which we didn't know anything about, and we made an artificial gene with a promoter and purified it in *E*. *coli [Escherichia Coli]*. When RNA transcribed in vitro from the artificial gene underwent splicing, then we were convinced.

During that year, there was a slow transition from thinking there was a protein to thinking there couldn't be one. By the time we did the final experiment, it had to work without the protein because there was no backup plan.

**Gitschier:** Have you had any other “eureka” moments?

**Cech:** We've been fortunate to have a couple more, but they weren't as exciting to me because I was doing those [splicing] experiments with my own hands, together with Arthur Zaug and Paula Grabowski. That is a special kind of “eureka” moment.

Since then, we've had a couple of big moments in the lab. One was finding the catalytic subunit of telomerase, something that had been predicted by Liz Blackburn and Carol Greider as early as 1985, when they described telomerase and found the RNA subunit. But ten years had gone by, and the whole world and a couple of biotech companies were searching for the catalytic subunit, when a postdoc in my lab, Joachim Lingner, purified the catalytic subunit from euplotes. We called it TERT telomerase for “telomerase reverse transcriptase.”

And in 2001, Peter Baumann found a protein that caps off the very ends of human chromosomes; we called it POT1 for “protection of telomeres.” We found it first in fission yeast, and did the genetics there.

The human genome project is great—we do all this work in simple organisms, and if you believe in evolution, you believe these things are probably widespread and can be found in the human database. That was another very exciting moment, again, because these telomere-capping proteins had been found 20 years earlier in ciliates, and people had been wondering whether the ciliates were special or whether all eukaryotes would have them.

**Gitschier:** Were you disappointed that these weren't RNA molecules?

**Cech:** Well, it was exciting enough that telomerase was half RNA.

**Gitschier:** Can you speak about how our whole perspective has changed in the last 20 years since the discovery of RNA as a catalytic moiety?

**Cech:** There are so many areas in which the RNA science has provided excitement. The whole nature of the ribosome, for example, and the work in Harry Noller's lab at UCSC [University of California Santa Cruz]—where his experiments indicated very strongly that RNA in the ribosome was doing more than just scaffolding key proteins, and that the RNA was the important entity for peptidyl transfer. That work came to its culmination when Tom Steitz and Peter Moore determined the crystal structure of the large subunit of the ribosome, showing that at the peptidyl transfer center, there was only RNA.

But look back at Jacob and Monod's early thinking that the lac repressor might be an RNA molecule. Once the lac repressor was proven to be a protein, people got all focused on gene expression being regulated by proteins. But if they had been looking in bacillus, rather than in *E*. *coli,* they would have found riboswitches built into transcripts that bind small-molecule metabolites and control transcriptional termination or translation. So the old Jacob/Monod model was actually right, but for a different branch of bacteria.

And of course, you can't even turn around without seeing RNAi [RNA interference] and siRNA [small interfering RNA] all over the place. So chances are there are still a lot of RNA-level functions to be discovered in complex genomes.

**Gitschier:** In an interview, you said you were a prolific writer as a younger man. What kind of writing did you enjoy, and do you still write?

**Cech:** I don't know that I ever enjoyed writing—writing is painful!

I started with a fourth grade teacher who made us write essays till our hands felt like they would fall off. I did a lot of writing at Grinnell College—humanities courses, great books courses, and a constitutional history course. I think learning to write a good argument and support a point of view in the humanities really helps your scientific writing. Scientific writing is particularly difficult, and you need all the help you can get. I've never written fiction or poetry or anything really interesting.

**Gitschier:** Do you ever think about attempting that?

**Cech:** I do think often about writing a book about my own experience in science. I like these books that some of my colleagues have written because they humanize the process, and they show how twisted the path to scientific knowledge is. I think there is so little understanding in the general public about the life of a scientist or the scientific process, and especially about the intense skepticism that we bring to everything we hear about or to even the discoveries in our own lab. I think if more of the problems in politics, governmental policy, and international affairs were approached with more of a scientific attitude, we would all be better off.

**Gitschier:** I would agree with you, and I would encourage you to follow through on that. What have been some of your favorites?

**Cech:** I re-read Watson's *Double Helix* recently. It's really well written: the excitement, the uncertainty, the despair and disappointment, and the exhilaration. That's probably the best example of a scientist giving an account of a portion of his own work.

**Gitschier:** You love doing research, and you're obviously so good at it, so why did you take the job of president of the HHMI?

**Cech:** After the Nobel Prize, I had a great decade of additional work and a big lab of 25 or more students and postdocs, who are now professors at good places around the country. And I did a lot of teaching; for instance, I insisted on teaching general chemistry for freshman for six years.

But I felt I was making an impact on only a local level, and I had a need to make an impact more nationally. So I started to serve on some scientific advisory boards. It was useful; however, I'd go there and have a great interaction, but then I'd leave and the people [I was advising] were still stuck there. If you really want to nurture something, you have to be there when the initial excitement subsides.

So I thought I would consider moving into a leadership position, but promised my family I would wait till my daughters finished high school. Then the Hughes position came along and that seemed so special, and one that doesn't come along very often. It was the idea of having an impact on the educational programs that was the big driver for me. The investigator program was so well run that there wasn't so much of a challenge.

**Gitschier:** Max Cowan [former vice president of the HHMI] told me that you were one of his top choices for the HHMI presidency, specifically because of your keen commitment to teaching.

**Cech:** Is that right? Well, thank you! He never shared that with me, although we always had a very good relationship.

We've evaluated all the educational programs; we've discontinued a number of them, started new ones, and massaged others. The role of a nonprofit is to do something different from the federal government, including things that have some risk, because you want to try something that can have a huge impact. But then you have to have the discipline and strength to discontinue things if they aren't successful.

Every time we've discontinued a program, there is a firestorm of outrage in the community, and I get accosted personally and get a lot of angry letters, but that's part of being in leadership—to evaluate and get good advice from the best people you can find, including the trustees, and then to just do it.

**Gitschier:** So, how do you like the job?

**Cech:** There are large parts that I find to be enjoyable, and there are also significant parts that I find important to be done well but which there's not much enjoyment in. You know, it's really wonderful being a university professor!

**Gitschier:** I know!

**Cech:** There is tremendous amount of freedom, and people don't yell at you very much either!

In this job, you make a lot of people happy and a lot of people very unhappy, and the people who you make unhappy are sometimes gracious and understanding, but sometimes they'll never forgive you. But you have a process, and you have to take the scientific review board advice extremely seriously. And it's also really important for there to be turnover.

**Gitschier:** Now that you are at the helm of the HHMI enterprise, you have a special vantage point overlooking biomedical research. What do you find the most interesting?

**Cech:** The most eye-opening for me has been the neurosciences, probably because it's the area where I was the most ignorant, but also because it's extremely exciting to be probing the cellular and molecular basis of behavior, sensory perception, and memory, and moving toward understanding consciousness and cognition. I find the neuroscience talks to be the most riveting.

The other area, also very far from my upbringing, that I find to be so gratifying and so important is translational medical research. People doing really high-quality basic research stimulated by what they have seen in the clinic. It is really exciting when one of the investigators makes a mouse model that recapitulates a human disease, treats the mouse, and gets an effect, and now is treating patients and gets remediation of the disease.

**Gitschier:** So if you were going to start a postdoc today, what would you choose?

**Cech:** I'd probably still be a boring chemist because I personally love thinking about the molecules. I do want to be thinking about molecules that are important for human health.

I think you have to do what your passion is. My passion is to think about nucleic acids folding up and interacting with themselves, interacting with small molecules, and interacting with proteins, and also to think about biochemical reactions, about how their specificity and rate enhancement is generated. In ten years, the stuff we're doing will probably be totally unfundable!

**Gitschier:** How long do you think you'll be staying with the HHMI?

**Cech:** I serve at the pleasure of the trustees, and so far they've been supportive. Things are still exciting. It's been very important for me to have been able to continue some research because it uses a completely different set of neurons than administrative work.

There is a search for absolute truth in research. You never get there—but there are criteria by which you judge how close you are. You're always criticizing yourself and criticizing your colleagues, and they're criticizing you. And there is a test, very often, that you can do to decide who's right.

But in administrative work, it's all about empowering people, and there is never any absolute truth, and you can never fool yourself into thinking you've made all the right decisions. They are completely different jobs, and I enjoy them both.

There is perceived value to Hughes to have the leadership actively engaged in science. The real question is what will happen to my 30-year NIH grant coming up for renewal next year. 

**Figure:**
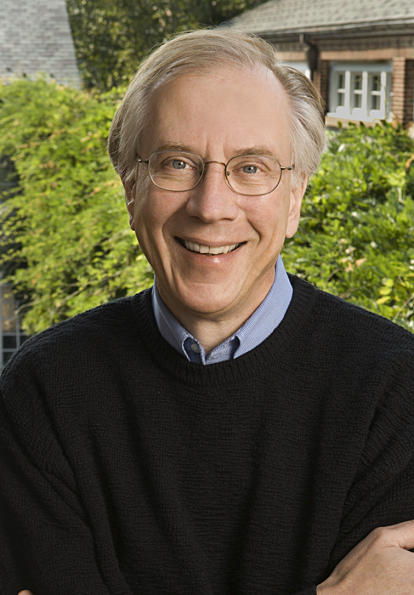
Tom Cech

